# Social vital signs for improving awareness about social determinants of health

**DOI:** 10.1002/jgf2.251

**Published:** 2019-05-02

**Authors:** Junki Mizumoto, Toshihiro Terui, Masanari Komatsu, Akira Ohya, Satoshi Suzuki, Saori Horo, Daisuke Sugihara, Yumi Otaka, Ai Ashino, Haruki Imura, Yukinori Harada, Kenta Sato

**Affiliations:** ^1^ Department of Family Practice Ehime Seikyou Hospital Ehime Japan; ^2^ Junior Resident Health‐Coop Watari Hospital Fukushima Japan; ^3^ Division of General Medicine Kagoshima seikyo hospital Kagoshima Japan; ^4^ Division of General Medicine Mimihara general hospital Osaka Japan; ^5^ Division of General Medicine Tone Chuo Hospital Gunma Japan; ^6^ Nursing Department Kin‐ikyo Sapporo Hospital Hokkaido Japan; ^7^ Socialwork Service Office Nagano Chuo Hospital Nagano Japan; ^8^ Department of General Medicine Kensei Hospital Aomori Japan; ^9^ Saitama Center for General and Family Medicine Saitama Japan; ^10^ Department of Health Informatics Kyoto University School of Public Health Kyoto Japan; ^11^ Department of General Medicine Amagasaki Medical Coop Hospital Hyogo Japan; ^12^ Department of Diagnostic and Generalist Medicine Dokkyo Medical University Hospital Tochigi Japan; ^13^ Department of General Medicine Kin‐ikyo Sapporo Hospital Hokkaido Japan

## Abstract

We, Team SAIL, have held sessions introducing social vital signs (SVS). SVS is a useful tool for evaluating patient’s social determinants of health (SDH).

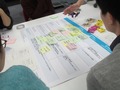

As primary care providers, we recognize the importance of clinical approach based on the Biopsychosocial model, to assure patient‐centeredness and remind us about health advocacy.^1^ However, a patients’ social history and background of his/her life is easily overlooked. It is partly because we are uncertain about how to address patients’ social issues in clinical practice.^2^

Team SAIL (Scope to upstream and Action with Interprofessional Investigating and Learning) was established in 2016 to develop an effective and easy‐to‐use tactics of addressing patients’ social determinants of health (SDH) and do research on related fields. The team consists of family physicians and residents, primary care nurses, medical social workers, clerks in clinic offices, and other health professionals. Our purpose is providing frameworks addressing the “causes of the causes,” or the underlying social factors leading to biomedical disturbance, of patients’ health issues,^3^ as if to sail “upstream” through a cautionary zone near the crest of a waterfall.^1^, ^4^

We have focused on and expanded the concept of social vital signs (SVS) so that primary care providers have more insight about SDH and higher sensitivity of their patients’ social problems. The term “SVS” was introduced in 2014 as an indicator of social characteristics which is easily measurable and broadly applicable.^5^ Our SVS is composed of 7 items, which mnemonic is “HEALTH + P.” “HEALTH + P” is our original framework and has been invented through literature review, interprofessional discussion, and clinical practice. This mnemonic is a tool to remind primary care providers of important questions to ask, and to encourage them to take a patients’ history related to micro and meso levels of SDH.

H: human network and relationships

E: employment and income

A: activities that make one's life worth living

L: literacy and learning environment

T: taking adequate food, shelter, and clothing

H: healthcare system

+P: patient preference and values

To date, we have held six workshops introducing SVS (Figure 1). Participants range widely from medical students, nursing students, doctors, nurses, pharmacists, social workers, and other professionals. Our workshops usually begin with brief introduction of SDH and the definition of terms to serve as a baseline. Then, we hold a case‐based small group work where participants apply the SVS. They summarize the patient's SVS in a chronological order by filling in a constructed chart, consider the “causes of the causes,” and discuss possible intervention strategies toward the patient's problems. The workshop ends with a large group discussion about options for introducing and implementing SVS in their local context. Through the workshop, we underline the importance of participants being aware of social determinants of their patients’ health． Among these sessions, the interprofessional discussion is especially significant because it provides participants an array of viewpoints and most of the real‐life cases require interprofessional assessments and interventions.

**Figure 1:**
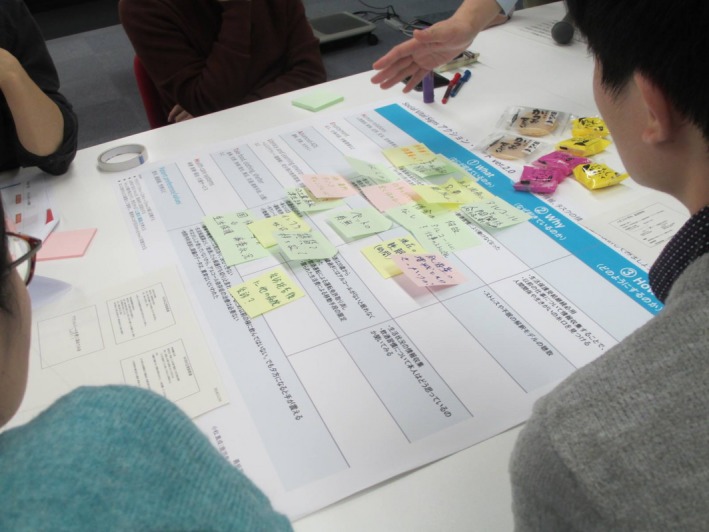
The view of our workshop. Participants are filling in constructed charts

Reports from participants submitted at each session reveal the following. Firstly, SVS helps recognize patients’ backgrounds and contexts at a glance, which is highly acclaimed by residents and young practitioners. Secondly, SVS may function as a lingua franca when members of an interprofessional team discuss patients’ problems. Thirdly, assessing SVS may avoid conceiving unnecessary negative emotions toward patients in trouble thus preventing excessive blame on personal responsibilities for patients’ health issues. In addition, more than half of participants in every workshop say they can fit SDH and SVS to their daily practice. Our future plans include the construction of theoretical frameworks and research on the effectiveness of applying SVS as an evaluation tool.

## CONFLICT OF INTEREST

The authors have stated explicitly that there are no conflicts of interest in connection with this article.

## References

[1] Rishi M . The upstream doctors: medical innovators track sickness to its source. TED Conference. 2013.

[2] Naz A , Rosenberg E , Andersson N , et al. Health workers who ask about social determinants of health are more likely to report helping patients: mixed‐methods study. Can Fam Physician. 2016;62(11):e684–e693. 28661888 PMC9844577

[3] Braveman P , Gottlieb L . The social determinants of health: it's time to consider the causes of the causes. Public Health Rep. 2014;129(suppl 2):19–31. 10.1177/00333549141291S206 PMC3863696 24385661

[4] Kiran T , Pinto AD . Swimming 'upstream' to tackle the social determinants of health. BMJ Qual Saf. 2016;25(3):138–40. 10.1136/bmjqs-2015-005008 26744423

[5] Institute of Medicine . Capturing social and behavioral domains and measures in electronic health records: phase 2. Washington, DC: The National Academies Press; 2014:227–36. 25590118

